# Organs Blood Flow during Elevation of Body Temperature in Sevoflurane Anesthetized Rats

**DOI:** 10.1155/2017/6182350

**Published:** 2017-06-04

**Authors:** Koichi Shimo, Ko Takakura, Kenji Shigemi

**Affiliations:** ^1^Department of Anesthesiology and Reanimatology, Faculty of Medical Sciences, Fukui University, Fukui, Japan; ^2^Department of Anesthesiology, Gifu Prefectural Gero Hot Spring Hospital, Gifu, Japan

## Abstract

The aim of this study is to investigate how elevation of body temperature changes organs blood flow during sevoflurane anesthesia. We conducted in vivo research on 14 male Wistar rats to monitor pulse rate and arterial blood pressure and measure hepatic, small intestinal, renal, and descending aortic blood flow using a laser Doppler blood flowmeter. We assessed the changes in organ blood flow, pulse rate, and arterial blood pressure during elevation of the rats' body temperatures up to 41.5°C under anesthesia with 2.0% or 3.0% sevoflurane. We concluded that elevation of body temperature up to 39.5°C does not change hepatic, small intestinal, and renal blood flow during 2.0 and 3.0% sevoflurane anesthesia.

## 1. Introduction

Perturbations in body temperature induce marked changes in heart rate, arterial blood pressure, and organ blood flow via sympathetic nervous activation. For example, high body temperature induces increases in splanchnic, renal, splenic, and lumbar sympathetic nerve activity [1, 2], resulting in constriction of the respective vascular beds and reduction in blood flow. In contrast, high body temperature elicits vasodilation of skin vasculature [3, 4]. Therefore, hyperthermia leads to redistribution of blood flow. Volatile anesthetics, for example, sevoflurane, also induce changes in organ blood flow [5] with decreasing sympathetic nervous activity [6].

There is little information on the overall effect of elevation of body temperature during general anesthesia with volatile anesthetics on organ blood flow. Therefore, the purpose of our study was to investigate how organ blood flow changes by elevated body temperature under volatile anesthetic using sevoflurane anesthesia. For this, we monitored hepatic, small intestinal, and renal blood flow during elevated body temperature in sevoflurane anesthetized rats using a laser Doppler blood flowmeter.

## 2. Methods

The experimental protocol was approved by the Institutional Animal Care Committee of Fukui University, Japan. Fourteen male Wistar rats (weight, 240–280 g) were used in this study.

The rats were anesthetized with 2.5% sevoflurane and their tracheas were cannulated, following which they breathed air and 2% sevoflurane with maintenance of spontaneous respiration. The left femoral artery was cannulated for monitoring arterial pressure and pulse rate and for blood collection. The left femoral vein was cannulated to administer saline (10 mL/kg/h) intravenously. Laparotomy was performed under local anesthesia with 1% lidocaine. A contact-type laser Doppler blood flowmeter FLO-C1 (Omegawave Inc., Tokyo) was used to monitor hepatic, small intestinal, and renal blood flow. Fiberglass probes JF1 connected to GJ probe (Omegawave Inc., Tokyo) were made to contact with the surface of the right side of the liver, the small intestine (3 cm distal to the duodenum), and the right kidney to monitor the blood flow through each of these organs at a depth of less than 1 mm below the organ surface [7, 8]. The blood flow in the descending aorta was estimated by monitoring with the probe inserted orally 4 cm into the esophagus where the esophagus contacts the descending aorta anatomically. A probe to measure body temperature was inserted 1 cm into the rectum via the anus.

After the surgical procedure and the local anesthesia, the rats were allowed to recover from them on a temperature-controlled mat for 1 hour under 2.0% sevoflurane anesthesia. The rats were then randomly assigned to receive either 2.0% or 3.0% sevoflurane (*n* = 7 each). The end-tidal sevoflurane concentration was monitored continuously using an infrared medical gas analyzer. Thirty minutes after the start of administration of the specified concentration of sevoflurane, arterial blood gases were measured in each animal (sample volume: 0.15 mL). Then, the body temperature was raised to 41.5°C using the temperature-controlled mat in approximately 60 min, and pulse rate, mean arterial blood pressure, and blood flow were monitored. It had been ascertained in a prior study that the study subjects were stable at 37.5°C for more than 90 min under both 2.0% and 3.0% sevoflurane anesthesia. Blood flow values were measured as milliliters per minute per 100 grams of tissue. The regional vascular resistance was calculated later by the mean arterial blood pressure divided by the regional organ blood flow. Arterial blood gases were measured again at a body temperature of 41.5°C.

### 2.1. Data Analysis

Data are expressed as mean (± SD). Differences between the respective values on pulse rate, arterial blood pressure, blood flow, and regional vascular resistance from 37.5 to 41.5°C were analyzed by the Kruskal-Wallis test, and the Steel method was used for post hoc multiple comparisons at 37.5°C (Excel Tokei 2008 software, SSRI Co, Tokyo, Japan). Differences between 37.5°C and 41.5°C arterial blood gas data were analyzed by Wilcoxon signed-rank test. Differences in pulse rate, mean arterial blood pressure, and blood flow between 2% and 3% sevoflurane data were analyzed by Mann–Whitney* U* test. For all the analyses, a* p* value of 0.05 was considered significant.

## 3. Results

Table 1 shows pulse rate, mean arterial blood pressure, and descending aortic, hepatic, renal, and small intestinal blood flow under 2.0% and 3.0% sevoflurane anesthesia at 37.5°C before elevation of body temperature. There was no significant difference between 2.0% and 3.0% sevoflurane anesthesia in pulse rate, mean arterial blood pressure, and each blood flow value.

**Figure 1:**
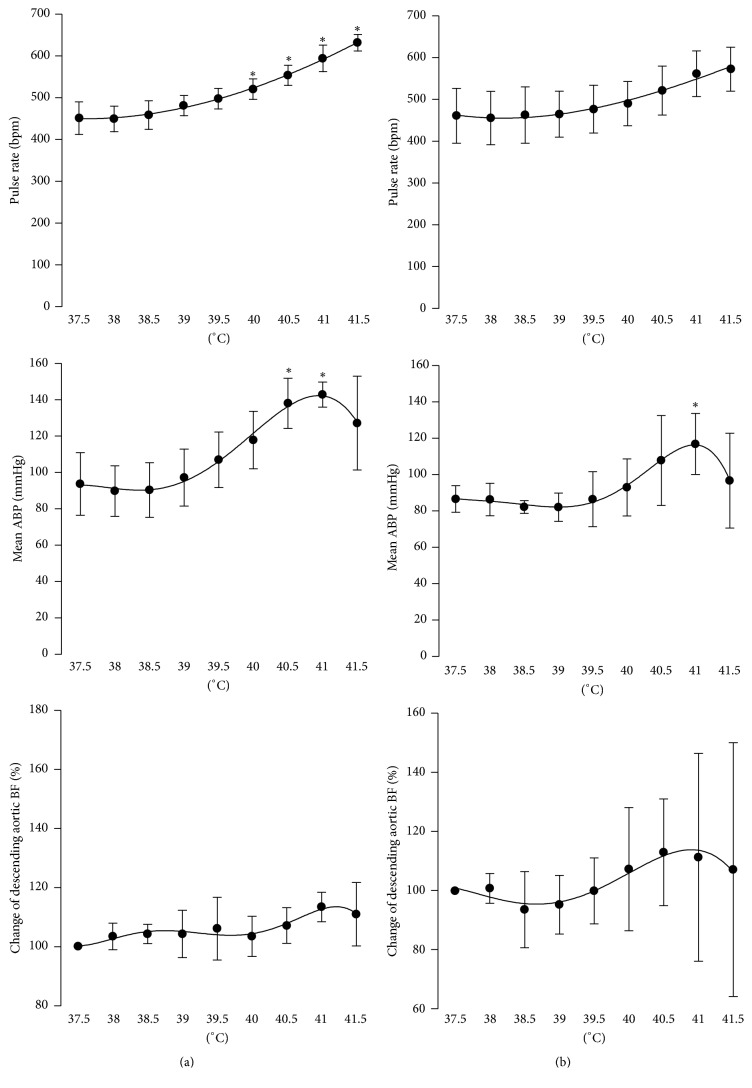
Changes in pulse rate, mean arterial blood pressure (ABP), and descending aortic blood flow by hyperthermia up to 41.5°C during 2.0% (a) or 3.0% (b) sevoflurane anesthesia.* n* = 7 each. Data are presented as mean ± SD. ^*∗*^Statistically significant differences versus the values at 37.5°C (by Steel method at a significance level of 0.05). BF: blood flow.

Pulse rate was maintained up to 39.5°C of body temperature under 2.0% sevoflurane anesthesia but increased thereafter (Figure 1). The increase did not appear under 3.0% sevoflurane anesthesia. Mean arterial pressure was also maintained up to around 40.0°C but increased thereafter with temperature elevation under both 2.0 and 3.0% sevoflurane.

**Figure 2:**
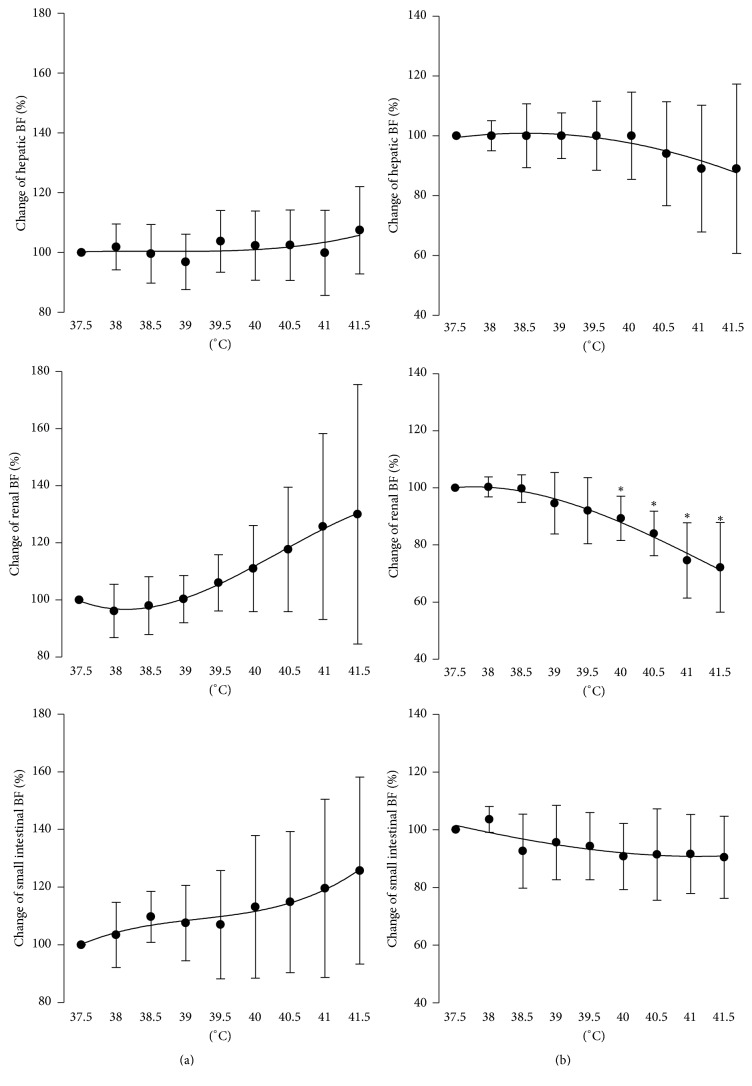
Changes in hepatic, renal, and small intestinal blood flow with hyperthermia up to 41.5°C during 2.0% (a) or 3.0% (b) sevoflurane anesthesia.* n* = 7 each. Data are presented as mean ± SD. ^*∗*^Statistically significant differences versus the values at 37.5°C (by Steel method at a significance level of 0.05). BF: blood flow.

Hepatic, renal, and small intestinal blood flows under 2.0% sevoflurane anesthesia were maintained during elevation of body temperature (Figure 2). Also under 3.0% sevoflurane, the blood flow was maintained during elevation of body temperature, but only renal blood flow decreased at over 40.0°C.

**Figure 3:**
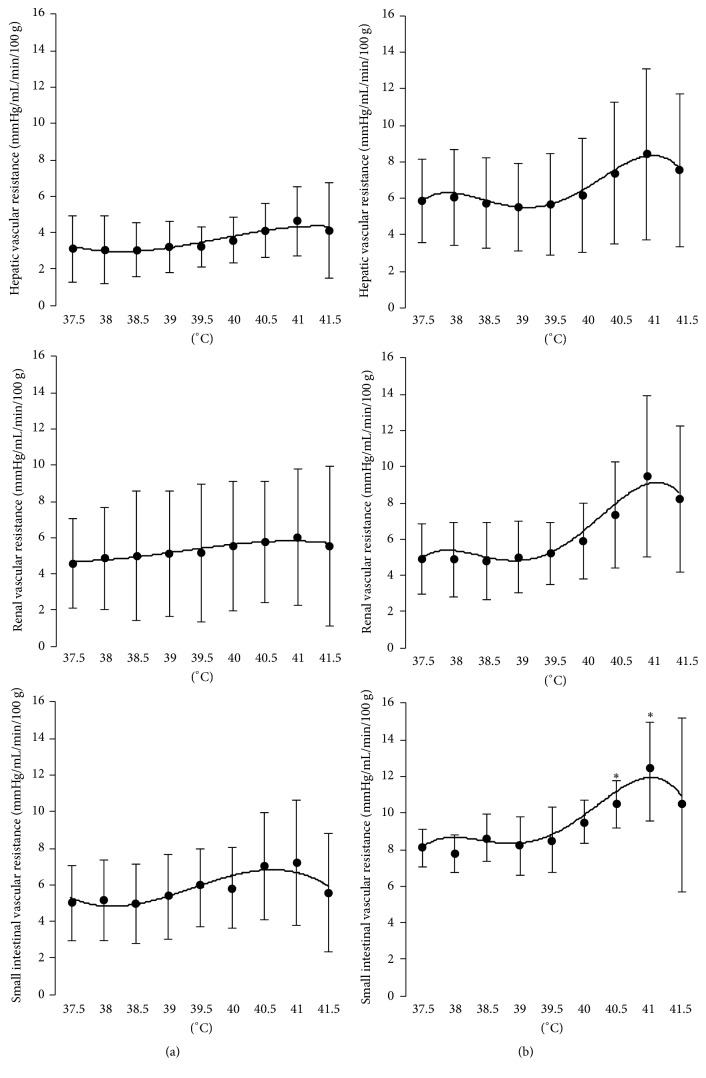
Changes in hepatic, renal, and small intestinal vascular resistance with hyperthermia up to 41.5°C during 2.0% (a) or 3.0% (b) sevoflurane anesthesia.* n* = 7 each. Data are presented as mean ± SD. ^*∗*^Statistically significant differences versus the values at 37.5°C (by Steel method at a significance level of 0.05).

The regional vascular resistance was almost unchanged in liver, kidney, and small intestine during elevation of body temperature under both 2.0 and 3.0% sevoflurane anesthesia (Figure 3).

Table 2 shows the results of arterial blood gas analysis during 2.0% and 3.0% sevoflurane anesthesia at 37.5 and 41.5°C. Base excess decreased after elevation of body temperature under both 2.0% and 3.0% sevoflurane anesthesia. PaCO_2_ and PaO_2_ did not change during hyperthermia under 2.0% sevoflurane anesthesia. However, both these values decreased significantly during hyperthermia under 3.0% sevoflurane anesthesia.

## 4. Discussion

The main finding of this study was that hepatic, small intestinal, and renal blood flows under 2.0 and 3.0% sevoflurane anesthesia were maintained during hyperthermia. Although only renal blood flow showed a significant decrease at over 40°C, the severe high temperature like this may be rarely encountered.

Hyperthermia induces an increase in splanchnic and renal sympathetic nervous activity [1, 2], resulting in vasoconstriction of the respective vascular beds. Therefore, a decrease in blood flow in the vascular beds is an expected natural result. However, contrary to this expectation, the decrease in the blood flow (Figure 2) and the increase in the vascular resistance (Figure 3) of the hepatic, small intestinal, and renal vascular beds due to hyperthermia were not seen under sevoflurane anesthesia, except 3% sevoflurane at over 40.0°C. As the studies mentioned above used chloralose as an anesthetic [1, 2], sevoflurane could inhibit the vasoconstriction induced by hyperthermia.

The hypothalamic paraventricular nucleus (PVN) seems to play a key role for decrease of the mesenteric and renal blood flow during hyperthermia [9, 10]. Nitric oxide and angiotensin II (acts on angiotensin 1A receptor) in the PVN are important in mediating the decrease of mesenteric and renal blood flow during hyperthermia [11, 12]. Sevoflurane does not affect the angiotensin 1A receptor signaling [13]. But it decreases neuronal nitric oxide synthase levels [14], which may be one of the reasons that sevoflurane could inhibit the vasoconstriction induced by hyperthermia. Although it is unknown whether chloralose affects nitric oxide synthase, it has been used as background anesthesia in many in vivo studies measuring neuronal nitric oxide synthase activity.

Regarding the direct effects of sevoflurane itself on blood flow, it maintains hepatic blood flow in both artificially ventilated and spontaneously breathing rats [15, 16]. Portal venous blood flow is also reportedly preserved with up to 1.0 MAC sevoflurane [17, 18]. Renal blood flow is unchanged with 1.0 MAC sevoflurane [15]. Less than 1.5 MAC sevoflurane also maintains small and large intestinal blood flow [16, 19]. Also in our study, sevoflurane-dose-dependent change in the organs blood flow was not observed (Table 1). Therefore, sevoflurane itself seems not to have direct effects on the organs blood flow.

## 5. Limitations

Many potential confounding variables can influence regional blood flow, including the type of animal species, type of ventilation used, body position, changes in arterial blood pressure, and arterial oxygen concentrations. We used rats that spontaneously breathed room air with sevoflurane. With this experimental protocol, PaCO_2_ and PaO_2_ decreased after hyperthermia under 3% sevoflurane anesthesia (Table 2). Although splanchnic and renal blood flows seem to be affected only at extremely low PaO_2_ and PaCO_2_ reportedly in rats [20, 21], it cannot be denied that the low PaCO_2_ and PaO_2_ after hyperthermia under 3% sevoflurane anesthesia in our study had any effect on the blood flow.

## 6. Conclusion

Hepatic, small intestinal, and renal blood flows in 2.0 and 3.0% sevoflurane anesthetized rats were maintained up to 39.5°C of body temperature.

## Figures and Tables

**Table 1:** Pulse rate, mean arterial blood pressure, and blood flow under sevoflurane anesthesia at 37.5°C before hyperthermia.

	2% sevoflurane	3% sevoflurane	*p*
Pulse rate (bpm)	450 (39)	458 (66)	0.953
Mean blood pressure (mmHg)	93 (17)	86 (7.4)	0.226
Descending aortic blood flow (mL/min/100 g)	49.9 (5.5)	40.0 (13.0)	0.159
Hepatic blood flow (mL/min/100 g)	34.4 (16.9)	20.1 (10.8)	0.085
Renal blood flow (mL/min/100 g)	28.4 (14.2)	22.5 (9.6)	0.521
Small intestinal blood flow (mL/min/100 g)	22.1 (7.3)	23.1 (16.3)	0.798

Mean (SD). *n* = 7 each. *p*: Mann–Whitney *U* test.

**Table 2:** Arterial blood gas analysis under sevoflurane anesthesia at 37.5 and 41.5°C.

	2% sevoflurane			3% sevoflurane		
	37.5°C	41.5°C	*p*	37.5°C	41.5°C	*p*
pH	7.42 (0.02)	7.38 (0.06)	0.306	7.39 (0.03)	7.37 (0.07)	0.481
PaCO_2_ (mmHg)	37 (2)	31 (6)	0.108	40 (6)	30 (3)	<0.01
PaO_2_ (mmHg)	71 (5)	61 (12)	0.309	73 (12)	51 (8)	0.012
Base excess	0.0 (0.6)	−5.9 (1.7)	<0.01	−0.6 (1.7)	−6.6 (4.2)	<0.01

Mean (SD). *n* = 7 each. *p*: Wilcoxon signed-rank test.
